# Bisoprolol Treatment and Adherence in Pediatric Patients With Genotype‐Positive Long QT Syndrome

**DOI:** 10.1002/joa3.70422

**Published:** 2026-07-14

**Authors:** Eemil Taipalus, Minna Mecklin, Antti Tikkakoski, Tuija Poutanen, Kaisa Ylänen

**Affiliations:** ^1^ Faculty of Medicine and Health Technology Tampere University Tampere Finland; ^2^ Department of Paediatrics, Wellbeing Services County of Pirkanmaa Tampere University Hospital Tampere Finland; ^3^ Faculty of Medicine and Health Technology, Tampere Center for Child, Adolescent and Maternal Health Research Tampere University Tampere Finland; ^4^ Department of Clinical Physiology and Nuclear Medicine, Wellbeing Services County of Pirkanmaa Tampere University Hospital Tampere Finland

**Keywords:** bisoprolol, genotype‐positive children, long QT syndrome (LQTS), pediatricsBeta‐blockers

## Abstract

**Background:**

Non‐selective beta‐blockers, such as propranolol, are highly effective in reducing arrhythmia risk in long QT Syndrome (LQTS). The study aim was to evaluate the use of beta‐blockers, especially beta‐1‐selective bisoprolol, and their efficacy in Finnish children with genotype‐positive LQTS type 1 or 2.

**Methods:**

We retrospectively reviewed the medical records of children with genetically confirmed LQTS1 or LQTS2 diagnosis, who were treated at Tampere University Hospital in Finland between 2006 and 2020. Data on cardiac events, beta‐blocker use and adherence, ECG, clinical exercise tests, and genetic testing were collected.

**Results:**

Eighty‐eight children were diagnosed with a genotype‐positive type 1 or 2 LQTS, and 84% of them had a family history of LQTS associated gene mutations. Four Finnish founder mutations accounted for 68% of the cases. At diagnosis, 63% had a normal QTc interval. No cardiac events occurred during follow‐up among patients receiving beta‐blocker therapy. For children aged 0–7 years, propranolol was the primary medication in 90% of cases, and for > 7‐year‐olds, bisoprolol was used in 77% of cases. At the last follow‐up visit, 87/88 were on beta‐blockers, most commonly bisoprolol (66%). Medication adherence was high, with 84% of patients using beta‐blockers as prescribed. In clinical exercise tests, maximal heart rate remained < 160 beats per minute in 76% of tests, indicating effective beta‐blockade.

**Conclusions:**

Cascade screening identified asymptomatic patients, most with normal QTc intervals. Bisoprolol was the most commonly used beta‐blocker with good adherence, effective heart rate control, and no cardiac events observed during follow‐up among these Finnish children with LQTS.

## Graphical abstract

LQTS is commonly treated with non‐selective beta‐blockers. In this study on Finnish pediatric patients with genotype positive LQTS1 or LQTS2, most with a Finnish founder mutation, the beta‐1‐selective bisoprolol was the most commonly used beta‐blocker with good adherence, effective heart rate control, and no cardiac events observed during follow‐up.

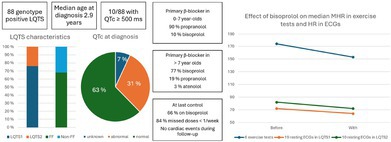

## 1 Introduction

Long QT Syndrome (LQTS) is an inherited cardiac arrhythmia disorder characterized by ventricular arrhythmias leading to syncope and even sudden cardiac death (SCD). Prevalence of LQTS is approximately 1:2000–1:2500 [1, 2, 3, 4, 5]. Beta‐blocker therapy is recommended in LQTS patients because they reduce arrhythmia‐related syncope and sudden death [1, 2, 3, 4, 5, 6, 7, 8]. The non‐selective beta‐blockers nadolol and propranolol have the strongest research evidence [4, 9, 10]. However, in clinical practice, different beta‐blockers are used for LQTS patients.

The most common disease‐causing genes in LQTS are KCNQ1, KCNH2, and SCN5A, corresponding to subtypes LQTS1, LQTS2, and LQTS3, respectively [4, 11]. LQTS types 1 and 2 are the most common subtypes with a similar therapeutic approach, whereas the rarer LQTS3 requires a more individualized treatment strategy. Four LQTS gene mutations show a higher prevalence in the Finnish population compared to other regions and are regarded as Finnish founder (FF) mutations [12, 13]. These FF mutations account for nearly 75% of gene‐positive LQTS patients in Finland [12]. KCNQ1 c.1766G > A p. (Gly589Asp) and KCNQ1 c.1129‐2A > G constitute FF mutations for LQTS1, and KCNH2 c.526C > T p. (Arg176Trp) and KCNH2 c.1655 T > C p. (Leu522Ser) for LQTS2. FF mutations have been reported to cause a milder QTc‐prolonging effect and fewer cardiac events than non‐founder variants in both pediatric and adult cohorts [14, 15]. Bazett's formula is widely used in Europe for QTc measurement, as many clinical guidelines and diagnostic thresholds are based on reference values derived using Bazett's correction [4, 6, 16, 17, 18, 19].

The European guidelines for the management of ventricular arrhythmias and the prevention of sudden cardiac death recommend beta‐blockers in all LQTS patients and note that relatives with a mutation but without QT prolongation are at risk of experiencing arrhythmia, although less frequently than phenotype‐positive patients [4]. Other guidelines also recommend beta‐blocker medication to all LQTS patients, with or without QTc prolongation [19, 20]. Importantly, evidence regarding intentional non‐therapy in LQTS is limited, particularly in pediatric populations [21]. Risk assessment in children is challenging, as symptom reporting and co‐operation during diagnostic testing may differ significantly from adults. Consequently, in Finland, it is a common clinical practice to initiate beta‐blocker therapy in all children with LQTS regardless of the QTc interval.

In Finland, propranolol and bisoprolol are the most used beta‐blockers for treating LQTS in children as nadolol is not available in Finland [14, 22]. Propranolol has the strongest evidence, but bisoprolol is also used in clinical practice in adult and pediatric patients. Bisoprolol is used in patients considered to be at a relatively lower risk of cardiac events, reflecting national treatment practices despite its beta‐1 selectivity [20]. Propranolol is commonly prescribed for preschool‐aged children, whereas bisoprolol is often preferred for older children due to its possibility of once‐daily dosing. Once‐daily dosing can factor into medication adherence, which is known to be a major factor in risk assessment [23, 24]. Long‐acting propranolol is available only with a special license and in adult tablet strengths (80 and 160 mg), making accurate dosing in small children challenging. Thus, bisoprolol has become a practical nationwide alternative because it is available in several generic forms. Although data on bisoprolol use in LQTS is limited [9, 25, 26], it has shown good pharmacological predictability in adults [27]. Its excellent availability is also one of the factors favoring bisoprolol in Finland. Evidence for atenolol in LQTS is also limited and mainly concerns LQTS1 [9, 28].

The aim of the study was to evaluate the use, adherence, and therapeutic response to beta‐blocker therapy, especially beta‐1‐selective bisoprolol, in Finnish children with genotype‐positive LQTS1 or LQTS2, most of whom have milder forms of LQTS variants.

## 2 Materials and Methods

We retrospectively reviewed the patient charts of children diagnosed with LQTS, who were treated at Tampere University Hospital Pediatric Cardiology Outpatient Clinic between 1.1.2006–31.12.2020. Patients with LQTS type 3 were excluded from the study. The patient population was divided into two age groups (0–7 and > 7 years old), based on clinical, diagnostic and medication practice.

All data, including information regarding treatment and symptoms, genetic test results, ECGs and clinical exercise tests were retrospectively collected from the patient records. Documented arrhythmias, LQTS‐related syncope, implantable cardioverter‐defibrillator (ICD) therapy, aborted cardiac arrest or SCD were considered cardiac events. The standard protocol for patient visits includes, in addition to a clinical examination and ECG, an interview regarding medication, adherence, and symptoms, all of which are documented in the patient records. Medication adherence was assessed at each clinical control based on self‐report and documented in the patient records. Data were collected from clinical control visits at the time of diagnosis, after the initiation of beta‐blocker therapy, at the first follow‐up visit, and at the last follow‐up visit (either at the age of 16–18 years or during 2021–2022).

The ECGs were analyzed at the time of each visit. The QTc intervals were manually measured and calculated according to Bazett's formula. In a standard 12‐lead resting ECG, a QTc interval > 460 ms was considered abnormal for females and > 450 ms for males [29]. Clinical exercise test data were collected if the test was conducted at that clinical control. Maximum heart rate was calculated using the formula 205–½ × age. A clinical exercise test was performed using a bicycle ergometer for school‐aged children. It is standard protocol to encourage the child to achieve maximal effort. Younger patients were not tested due to limited co‐operation and small body size. A maximum heart rate < 160 beats per minute (BPM) was interpreted as an adequate beta‐blocker response and served as an additional marker of medication adherence.

LQTS subtypes (LQTS1 and LQTS2) were defined based on the genetic defect [11]. KCNQ1 c.1766G > A p. (Gly589Asp) and KCNQ1 c.1129‐2A > G constituted Finnish founder (FF) mutations for LQTS1, and KCNH2 c.526C > T p. (Arg176Trp) and KCNH2 c.1655 T > C p. (Leu522Ser) for LQT2 [12, 13]. In cascade screening, the known familial mutation was analyzed. In patients without a family history of LQTS, genetic testing was performed using broader gene panels. Prior to 2012, it was common practice to first use a gene panel targeting the four FF mutations. After 2012, broader gene panels covering a wider range of established LQTS‐associated mutations were introduced and have since been used.

Statistical analyses were performed using SPSS version 30.0.0.0. Frequencies and percentages were used for categorical variables. The median and range were expressed for the non‐normally distributed variables. Categorical variables were compared with the Pearson chi‐square test. For two‐group comparisons, the Mann–Whitney U test was used for nonparametric variables. The paired samples were analyzed using the Wilcoxon signed‐rank test. Statistical significance level was defined as (two‐sided) *p* < 0.05.

The Research Director of the Pirkanmaa Hospital District gave permission for the study. According to Finnish legislation, register‐based studies do not need Ethics Committee approval.

## 3 Results

A total of 127 patients with clinical suspicion of LQTS were followed at Pediatric Cardiology Outpatient Clinic of Tampere University Hospital between 2006 and 2020. Twenty‐seven were excluded due to an alternative arrhythmia diagnosis or diagnostic uncertainty (e.g., variants of uncertain significance without clinical findings of LQTS). Additionally, 11 patients with negative genetic testing and one patient with an LQTS3 diagnosis were excluded.

The final study cohort comprised 88 children with genotype‐positive LQTS of whom 67 (76%) had LQTS1 and 21 (24%) had LQTS2. The four FF mutations constituted 60 (68%) out of all cases (Table 1). All patients had genetically confirmed LQTS, identified either through primary family testing in 74 cases, or through broader panels in the remaining 14 patients without a family history of LQTS. Of these 14 patients, eight were diagnosed using broader gene panels and six using FF mutation panels. In two patients, two different genetic panels were conducted. Diagnoses were made between 1992 and 2020. Half of the patients (44/88) were female. The median age at diagnosis was 2.9 years (range: 0–17.6), age at last control was 14.0 years (0.1–17.6) and follow‐up time was 5.4 years (0–16.7). The diagnostic findings and beta‐blocker usage of the patients are described in Table 1.

**TABLE 1:** Findings at diagnosis, indications for cardiac evaluation, and use of beta‐blockers in the study population (*N* = 88).

	*N* = 88
Age at diagnosis, years	2.9 (0–17.6)
Sex, female, *n*	44 (50)
LQTS types, *n*	
LQTS1	67 (76)
LQTS2	21 (24)
LQTS1 mutation, *n*	
FF KCNQ1 c.1766G > A p. (Gly589Asp)	44 (50)
FF KCNQ1 c.1129‐2A > G	2 (2)
Non‐FF KCNQ1	21 (24)
LQTS2 mutation, *n*	
FF KCNH2 c.526C > T p. (Arg176Trp)	12 (14)
FF KCNH2 c.1655 T > C p. (Leu522Ser)	2 (2)
non‐FF KCNH2	7 (8)
QTc at time of diagnosis, ms	450 (370–550)
QTc at last control, ms	430 (360–500)
QTc normal at diagnosis, *n*	55 (63)
Indication for cardiac evaluation, *n*	
Cascade screening	74 (84)
ECG taken for non‐symptomatic reasons	9 (10)
Symptoms	5 (6)
Beta‐blocker started at diagnosis^1^, *n*	
Propranolol	50 (57)
Bisoprolol	27 (31)
Atenolol	1 (1)
Beta‐blocker at last follow‐up^2^, *n*	
Propranolol	27 (31)
Bisoprolol	58 (66)
Atenolol	2 (2)

*Note:* Values are presented as median (range) or *n* (%). Percentages are calculated as proportions of the total study population.

Abbreviation: FF, Finnish founder mutation.

^1^ *N* = 78 for beta‐blockers started at diagnosis.

^2^ *N* = 87 for beta‐blocker users at the last follow‐up.

Five patients were symptomatic at diagnosis, and their QTc ranged from 450 to 520 ms. Three had LQTS1 (one with a non‐FF mutation), and two had LQTS2 (both non‐FF mutations). Reported symptoms included bradycardia (*n* = 1), syncope (*n* = 1), palpitations (*n* = 3), and seizures (*n* = 1). No symptoms were reported during beta‐blocker treatment. For two of these patients, bisoprolol was the primary beta‐blocker.

At the time of diagnosis, QTc was normal in 55 (63%) patients, abnormal in 27 (31%), and unavailable in 6 (7%). Among carriers of FF mutations, QTc was abnormal in 15 cases (25%) compared with 12 (43%) among patients with non‐founder mutations. The median QTc interval and the proportion of patients with QTc ≥ 500 ms did not differ between LQTS1 patients with FF mutations and non‐FF KCNQ1 mutations at diagnosis prior to beta‐blocker therapy: 450 ms (400–550) vs. 450 ms (370–500), *p* = 0.355 and 6/40 (15%) vs. 1/21 (5%), *p* = 0.233, respectively. In total, 10 patients (5 females, 5 males) had QTc ≥ 500 ms at the time of diagnosis. QTc at diagnosis did not differ between females and males: 450 ms (370–520) vs. 450 ms (370–550), *p* = 0.362.

Beta‐blocker therapy was initiated in 78 (89%) of patients at the time of LQTS diagnosis (Table 1), proportions between the subgroups LQTS1 59 (88%) and LQTS2 19 (90%) did not differ. Nine patients started beta‐blocker therapy later either due to more precise information about the genetic mutation or changes in family preferences. The only patient not receiving beta‐blocker therapy carried an asymptomatic FF mutation KCNH2 c.526C > T p. (Arg176Trp) and was followed without medication as long as QTc remained within normal limits. No cardiac events were reported during the follow‐up while receiving beta‐blocker therapy, although seven patients reported palpitations without documented arrhythmia.

At diagnosis, propranolol was the first beta‐blocker with 50 (57%) and bisoprolol with 27 (31%) patients; no patients received long‐acting propranolol (Table 1). The most common beta‐blockers initiated at any time during follow‐up were propranolol in 90% of children younger than 7 years, whereas bisoprolol was initiated in 77% of children older than 7 years; age at treatment initiation was unavailable for 4 patients (Table 2). At the last follow‐up, 44 patients were aged 14 years or older. Among them, 37 (84%) used bisoprolol, 5 (11%) propranolol, and 2 (5%) atenolol. During follow‐up, beta‐blocker medication was changed in 26 (29%) patients, most often from propranolol to bisoprolol in 23 (88%) cases. The remaining 3 (12%) switched from atenolol to bisoprolol after earlier treatment with propranolol. The median age at the time of beta‐blocker change was 8 years (3–16). The most common reason for switching was a change to bisoprolol at school‐age to make dosing easier (*n* = 17). Other reasons included family preference (*n* = 4), dosing convenience later in school‐age (*n* = 2), side effects (*n* = 2), and insufficient adherence (*n* = 1).

**TABLE 2:** First beta‐blocker started by age groups (*N* = 83).

	Propranolol	Bisoprolol	Atenolol
0–7 years^1^, *n*	47 (90)	5 (10)	0
> 7 years^2^, *n*	6 (19)	24 (77)	1 (3)

*Note:* *N* = 83 for patients with known ages at initiation of beta‐blocker therapy. Values are presented as *n* (%). Percentages calculated as proportions of the corresponding age group.

^1^ *n* = 52 for beta‐blockers started in the age group.

^2^ *n* = 31 for beta‐blockers started in the age group.

At the last follow‐up visit, the median beta‐blocker dose was 0.10 mg/kg (0.05–0.13) for bisoprolol, 2.50 mg/kg (1.61–3.14) for propranolol, and 1.17 mg/kg for atenolol. There were no reported hypoglycaemias that led to hospitalization or required medication changes. At the last control, 73/87 (84%) of patients reported missing doses less than once a week.

In total, 63 exercise tests were performed during beta‐blocker therapy in 47 patients, and maximal heart rate remained < 160/BPM in 48 (76%) of the assessments. The beta‐blocker response in these tests is presented in Table 3.

**TABLE 3:** Beta‐blocker response in clinical exercise tests (*N* = 63) in 47 patients diagnosed with LQTS.

	All tests (*n* = 63)	Bisoprolol (*n* = 53)	Propranolol (*n* = 9)	Atenolol (*n* = 1)
MHR, BPM	150 (122–187)	150 (122–187)	141 (123–150)	181
MHR‐%	75 (61–94)	76 (62–94)	71 (61–74)	91
MHR < 160 BPM, *n*	48 (76)	39 (74)	9 (100)	0

*Note:* Values are presented as median (range) or *n* (%).

Abbreviations: BPM, beats per minute; MHR, maximum heart rate; MHR‐%, age‐appropriate maximum heart rate percentage.

**FIGURE 1:**
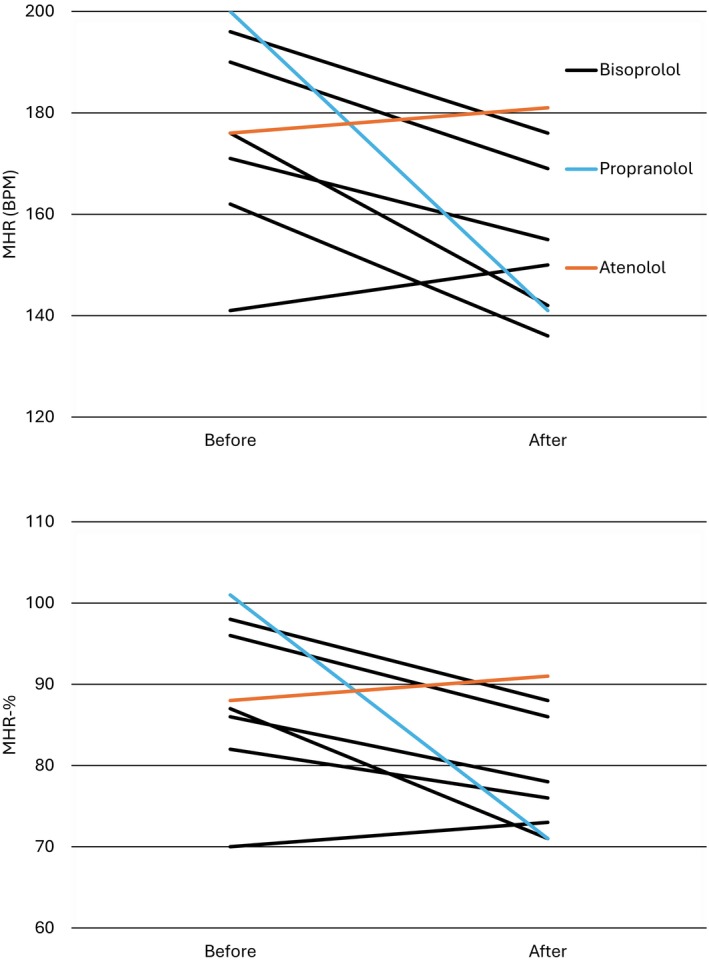
Median MHR and MHR‐% in clinical exercise tests before and after medication (*N* = 8). Clinical exercise test was performed before and after the initiation of medication in 6 patients with bisoprolol, 1 with propranolol, and 1 with atenolol. Abbreviations: BPM beats per minute; MHR, maximum heart rate; MHR‐%, age‐appropriate maximum heart rate percentage.

In six patients an exercise test was performed both before and after the initiation of bisoprolol. The median MHR decreased from 174 BPM (141–196) with an MHR‐percentage of 87% (70–98) to 153 BPM (136–176) with an MHR‐percentage of 77% (71–88), although these changes were not statistically significant (MHR *p* = 0.063 and MHR‐% *p* = 0.063). For both propranolol and atenolol, one patient underwent exercise testing before and after medication. With propranolol, MHR decreased from 200 to 141 BPM and MHR‐percentage from 101% to 71%, whereas with atenolol, MHR changed from 176 to 181 BMP and MHR‐percentage from 88% to 91%. Median MHR and MHR‐% values in these exercise tests are shown in Figure 1.

Total of 32 patients started bisoprolol as the primary therapy at diagnosis or during follow up. Baseline ECG data were unavailable for three patients who transferred into the hospital district. The effect of bisoprolol on resting ECG parameters by LQTS subgroups is shown in Table 4. Bisoprolol was associated with a significant reduction in heart rate in both LQTS1 and LQTS2 subgroups (*p* < 0.001 and 0.008, respectively), which was reflected in the QT interval shortening; however, QTc intervals did not change significantly (Table 4).

**TABLE 4:** Resting ECG findings before and during bisoprolol treatment by LQTS subgroup.

	LQTS1 (*n* = 19)			LQTS2 (*n* = 10)		
	Without bisoprolol	With bisoprolol	*p*	Without bisorpolol	With bisoprolol	*p*
HR (BPM)	72 (56–108)	64 (45–84)	< 0.001	82 (69–100)	72 (55–79)	0.008
PQ (ms)	140 (120–180)	140 (110–170)	0.586	140 (110–150)	140 (110–170)	0.861
QT (ms)	400 (360–470)	430 (360–490)	0.011	360 (320–460)	400 (360–480)	0.008
QTc (ms)	450 (420–510)	450 (410–500)	0.323	450 (370–550)	440 (390–630)	0.507

*Note:* Values are presented as median (range).

Abbreviations: BPM, beats per minute; HR, heart rate.

## 4 Discussion

This retrospective study showed that the majority of Finnish pediatric patients with LQTS type 1 or 2 remained symptom‐free both before diagnosis and during beta‐blocker medication. Most children younger than school age started beta‐blocker therapy with propranolol, whereas bisoprolol was the most common initial beta‐blocker in older children. Adherence to beta‐blockers appeared good, based on clinical history and maximum heart rate during exercise testing. Family cascade screening was the main indication for cardiac evaluation and led to the identification of many asymptomatic genotype‐positive children with normal QTc who might have otherwise remained undiagnosed.

In our cohort, short‐acting propranolol was the most common beta‐blocker started in children under school‐age. More than two‐thirds of school‐aged patients in our cohort initiated bisoprolol, and all beta‐blocker changes (*n* = 26) during follow‐up were from another medicine to bisoprolol. There is limited data on the use of bisoprolol in the treatment of LQTS. In a small cohort of 34 patients, more than half of whom lacked a genetic diagnosis, no significant cardiac event was observed during bisoprolol treatment [25]. In another study, although bisoprolol was found to shorten the QT interval in gene‐positive LQTS patients and was well tolerated, its anti‐arrhythmic efficacy compared to established beta‐blockers remained unclear [26].

Beta‐blockers are recommended for all LQTS patients, including children, because they reduce the risk of cardiac events [1, 2, 3, 4, 5, 6, 7, 8]. No cardiac events occurred in our patient cohort, in which 89% initiated beta‐blocker therapy at the time of LQTS diagnosis, and almost all were receiving beta‐blockers at later follow‐up. Although most patients in our cohort had a normal QTc at diagnosis and most were asymptomatic, adherence to beta‐blocker therapy was high, and no significant adverse effects were reported.

Medication adherence is one of the most important factors in preventing cardiac events in adult and pediatric patients alike [23, 24]. Heart rate on resting ECG or maximum heart rate during an exercise test can provide additional information on whether beta‐blockers are being taken as prescribed. The beta‐blocker dose has been shown to correlate with predicted peak heart rate in exercise testing [30]. In our study, a maximum heart rate below 160 BPM was defined as an indicator of adequate beta‐blocker response, and this was achieved in 76% of the 63 clinical exercise tests performed on 47 patients during therapy. This is consistent with the finding that 84% of patients reported missing doses less than once a week. In addition, bisoprolol effectively lowered the resting heart rate in 29 patients, further supporting both medication adherence and treatment efficacy. Exercise testing before and after starting bisoprolol was available in six patients. Although the sample size is very small, and the change did not reach statistical significance, the median percentage of predicted maximum heart rate decreased by 10%, warranting further study in larger cohorts.

Cultural, healthcare system and patient‐related factors all have a role in medication adherence [31]. In a study identifying medication adherence indicators in European countries, factors such as healthcare financing, e‐recipes and socio‐economic factors were identified as meaningful components [32]. Unified therapeutic strategies, accessible public healthcare and e‐recipes can all be thought of as having an impact on the good adherence findings. It is also a common counseling practice to ask about medication use and to renew the prescriptions routinely during clinical follow‐up visits. Previous studies have reported lower medication adherence. In a Northern Irish pediatric cohort, at least 57% had adequate medication adherence [33]. A similar New Zealand series reported 49% adequate adherence in mixed adult and pediatric patients [34]. In a Danish study of 633 adult patients, over one third had at least one treatment interruption longer than 60 days [35].

In our study, the selective beta‐blocker bisoprolol was well tolerated and showed good clinical response. This may reflect both drug efficacy and an underlying low‐risk status, and some patients might have remained symptom‐free even without medication. As knowledge about LQTS genotypes and phenotypes continues to accumulate, more individualized precision therapies may become possible in the future. Non‐treatment has been evaluated in adults with FF mutations and also in non‐founder populations, suggesting that carefully selected (e.g., asymptomatic individuals with normal QTc and certain low‐risk variants) patients can be followed without a major increase in event risk [15, 21, 36]. While strict adherence to beta‐blocker therapy is essential for high‐risk patients, selected low‐risk individuals might be managed with lifestyle counseling alone, such as avoidance of QT‐prolonging drugs. In a large register study of 3386 genotyped subjects, the cumulative probability of life‐threatening cardiac events from birth to age 40, was highest (15%) in patients with LQTS and prolonged QTc intervals, compared with 4% in mutation carriers with normal‐range QTc intervals and only 0.4% in unaffected family members [36]. Our findings, together with previous reports, suggest that certain FF mutations maybe safely managed without medication; however, further research is needed [15].

Due to extensive evidence, non‐selective beta‐blockers remain the drug of choice for patients with more malignant genotypes and in other higher risk situations, such as QTc exceeding 500 ms, symptomatic patients, or compound mutations [37]. Evidence on the use of bisoprolol in children with LQTS is still limited as well as in adult populations, even though bisoprolol is used to some extent [14, 33, 35].

In our study population, 63% of LQTS patients had a normal QTc interval at diagnosis, reflecting the known incomplete penetrance of LQTS where gene‐positive individuals remain asymptomatic with normal QTc intervals [6, 11, 38]. This proportion is higher than previously reported (36%–42%) [38, 39], which may partly be explained by the differences in QTc cut‐off values and the high prevalence of FF LQTS mutations, known to cause less pronounced QTc‐prolongation [14]. Possibly due to limited sample size, we did not detect significant differences in median QTc or in the proportion of patients with QTc ≥ 500 ms between LQT1 patients with or without an FF mutation.

This descriptive study provides valuable insight into LQTS in the Finnish pediatric population. This was a single‐centre retrospective study, with a relatively small sample size, and the generalizability of the results should therefore be interpreted with caution. The strength of the study is the availability of detailed clinical, ECG, clinical exercise test, and genetic data. Bisoprolol was used in a relatively large proportion of patients, offering valuable information on its use in this setting. Despite these limitations, the study adds to the limited evidence on bisoprolol treatment in children with LQTS.

## 5 Conclusions

Most children with LQTS in this Finnish cohort remained asymptomatic during follow‐up while on beta‐blocker therapy. Bisoprolol appeared to be an effective and well‐tolerated treatment option for LQTS in a population of gene variants with mild phenotypes. Once‐daily dosing may be a significant factor influencing medication adherence. Further research is needed to determine whether some of the asymptomatic FF mutation carriers with a normal QTc interval can be safely managed without beta‐blocker therapy. Bisoprolol still remains scarcely studied, and future research should focus on individualized risk assessment to determine which clinical factors are associated with a favorable response to bisoprolol treatment.

## Conflicts of Interest

The authors declare no conflicts of interest.

## Data Availability

Research data are not shared.
